# Change of physical activity parameters of hip and pelvic fracture patients during inpatient rehabilitation and after discharge: analysis of global and in-depth parameters

**DOI:** 10.1186/s11556-021-00261-1

**Published:** 2021-06-12

**Authors:** Karin Kampe, Klaus Pfeiffer, Ulrich Lindemann, Daniel Schoene, Kristin Taraldsen, Kilian Rapp, Clemens Becker, Jochen Klenk

**Affiliations:** 1 Department of Clinical Gerontology, Robert-Bosch-Hospital, Auerbachstr. 110, 70376 Stuttgart, Germany; 2 Institute of Medical Physics, Friedrich-Alexander-University Erlangen-Nürnberg, Henkestr. 91, 91052 Erlangen, Germany; 3 Department of Neuromedicine and Movement Science, Faculty of Medicine and Health Sciences, Norwegian University of Science and Technology, N-7491 Trondheim, Norway; 4 Institute of Epidemiology and Medical Biometry, Ulm University, Helmholtzstr. 22, 89081 Ulm, Germany; 5 IB University for Health and Social Sciences, Study Center Stuttgart, Paulinenstraße 45, 70178 Stuttgart, Germany

**Keywords:** Hip fracture, Pelvic fracture, Physical activity, Mobility, Older people, Digital mobility outcomes, Body-worn sensors

## Abstract

**Background:**

A growing number of older adults suffer hip and pelvic fractures leading to hospital admission. They often result in reduced physical activity (PA) and impaired mobility. PA can be objectively measured with body-worn sensors. Usually, global cumulative PA parameters are analysed, such as walking duration, upright-time and number of steps. These traditional parameters mix different domains of PA, such as physical capacity (PC), behaviour and living environment. We examined the change of global cumulative PA measures during rehabilitation and after discharge in patients with hip or pelvic fracture and whether more ‘in-depth’ PA parameters, such as walking interval length, variability of interval length and sit-to-stand transitions and their changes during rehabilitation and 3 months after discharge might better reflect the above mentioned three clinically relevant domains of PA.

**Methods:**

This study is a secondary data analysis of a randomised controlled trial to improve PA and fall-related self-efficacy in hip or pelvic fracture patients (≥60 years) with concerns about falling. Changes of accelerometer-measured global cumulative and in-depth PA parameters (activPAL3) were analysed in an observational design before and after discharge combining both groups. For comparison, the same analyses were applied to the traditional PC measures gait speed and 5-chair-rise.

**Results:**

Seventy-five percent of the 111 study participants were female (mean age: 82.5 (SD = 6.76) years. Daily walking duration, upright time and number of steps as aspects of global PA increased during inpatient rehabilitation as well as afterwards. The in-depth PA parameters showed differing patterns. While the total number of walking bouts increased similarly, the number of longer walking bouts decreased by 50% after discharge. This pattern was also seen for the average walking interval length, which increased by 2.34 s (95% confidence interval (CI): 0.68; 4.00) during inpatient rehabilitation and decreased afterwards below baseline level (− 4.19 s (95% CI: − 5.56; − 2.82)). The traditional PC measures showed similar patterns to the in-depth PA parameters with improvements during rehabilitation, but not at home.

**Conclusion:**

Our findings suggest that the in-depth PA parameters add further information to the global cumulative PA parameters. Whereas global cumulative PA parameters improved significantly during inpatient rehabilitation and after discharge, in-depth PA parameters as well as PC did not continuously improve at home. In contrast to global cumulative PA parameters the in-depth parameters seem to reflect contextual factors such as the build environment and aspects of PC, which are traditionally assessed by clinical PC measures. These in combination with digital mobility measures can help clinicians to assess the health status of fragility fracture patients, individually tailor therapy measures and monitor the rehabilitation process.

## Background

A growing number of older adults suffer hip and pelvic fractures occurring as a result of a low energy trauma [1]. In Europe alone, hip and pelvic fractures account for more than 1 million hospital admissions per year. These fractures are often associated with poor health outcomes, such as impaired mobility [2], disability, loss of independence [3], care home admissions [4] and increased mortality. Patients with pre-existing or post-fracture symptoms of anxiety, fear of falling, cognitive impairment, neurovascular and neurodegenerative diseases or limited fall-related self-efficacy are particularly threatened by these negative consequences after the fracture [5, 6]. Older persons with fragility fractures typically have excessive sedentary behaviour and low levels of mobility in the acute and sub-acute phase as well as post-discharge [7].

Improving PA and especially mobility that allow a return to the place of origin must be considered as the core elements and the goals of the rehabilitation process for fracture patients [8]. Sufficient levels of PA and mobility are the prerequisites for an active role in the society as they are key to perform meaningful indoor and outdoor activities [9]. Furthermore, a physically active lifestyle has been shown important to prevent and manage numerous conditions [10–12]. From a patient, caregiver, and health care professional perspective the sit-to-stand transfer, being able to walk a certain distance such as going to a toilet, the perceived safety of walking and reaching a certain gait speed is relevant for safe mobility. The restitution of independent walking at least with an assistive device is one of the primary goals during rehabilitation after hip and pelvic fractures [13].

Therefore, it makes sense to assess and monitor the PA during rehabilitation and after discharge. This enables clinicians to personalise therapy, give face valid feedback to patients and not the least could allow health care funders to assess cost-effectiveness and cost-utility of different approaches.

Body-worn sensors that objectively and continuously collect data on body movements are increasingly being used augmenting self-report measures of patient reported PA. In fragility fracture patients, the few studies using sensor-based measures have analysed global cumulative PA parameters. Number of steps as a surrogate measure of PA has been used as an outcome after hip fracture [14–16]. Another relevant PA construct is the cumulative daily upright duration, summarising standing and walking, which represents the other side of the coin of sedentary time including lying or sitting [17, 18]. Mean upright times after hip fracture have been reported to be less than 60 min per day in acute care settings [7, 14, 19]. During inpatient rehabilitation the mean upright time increased moderately to 80 min/day [20]. For community-dwelling older persons with a mean age of 76 years, Klenk and colleagues [21] reported in a German longitudinal study a walking duration of 104 min per day, a daily uptime of 380 min and a sedentary duration of 1060 min per day.

However, such traditional cumulative measures reflect a mix of several PA domains. By PA domains we mean three factors: Physical capacity (PC), such as muscle strength, behavioural aspects and environmental aspects. By behavioural aspects we mean the influence of for example the social environment and intrinsic or extrinsic motivation on PA. By environmental aspects we mean the influence of for example room sizes, ward lengths, accessibility of the housing environment and outdoor environment and number of stairs on PA. Therefore, besides these global cumulative PA parameters, we additionally analysed more ‘in-depth’ PA parameters, such as the pattern of different walking bout intervals, the variability of the walking bouts, the number of sit-to-stand transitions and other features, because we think that they might help to better reflect these mentioned PA domains, such as PC and behavioural or environmental aspects.

Therefore, the aim of our analysis was to examine the change of global cumulative PA measures during rehabilitation and after discharge in patients with hip or pelvic fracture and whether more ‘in-depth’ PA parameters, such as walking interval length, variability of interval length and sit-to-stand transitions and their changes during rehabilitation and 3 months after discharge might better reflect the mentioned three clinically relevant domains of PA.

## Methods

### Subjects and design

The current study is a secondary, explorative data analysis of a randomised controlled trial that aimed to improve PA and fall-related self-efficacy in hip and pelvic fracture patients [22, 23]. Participants were recruited between April 2011 and December 2013 from one geriatric rehabilitation facility in the Southwest of Germany. All participants gave written informed consent and the study protocol was approved by the ethical committee of the local university (ref: 113/2011BO2). The study procedures and the development of the fear of falling screening have been described in detail elsewhere [22]. In brief, all patients with hip or pelvic fractures (ICD-10 S72.0, S72.1, S72.2, S32.1, S32.2, S32.3, S32.4, S32.5, S32.7, S32.8) were screened after admission. Inclusion criteria were (1) age ≥ 60 years and (2) concerns about falling. Exclusion criteria were delirium, short-sightedness despite vision aid (Snellen Index > 20/400) [24], severe mental diseases, such as schizophrenia, suicidality, acute psychosis, cognitive impairment assessed by the Short-Orientation-Memory-Concentration Test (SOMC > 10) [25], being resident of a nursing home, not being able to understand the German language, no access to a telephone, severe aphasia, certain medical conditions, such as palliative treatment for cancer or not residing and accessible with public transportation in the metropolitan area. Medical exclusion criteria were determined by the physician in charge. Other exclusion criteria were asked by the assessment team.

The primary analyses did not show statistically significant differences between IG and CG regarding PA outcomes [24]. Therefore, we combined both groups in the present analysis. We also performed all analyses of the current study for each group, which confirmed no significant differences between groups (data not shown).

### Physical activity measurement

All PA outcomes were measured using an inertial sensor including a tri-axial accelerometer (activPAL3™, PAL Technologies Ltd., Glasgow, UK). The device was attached on the midline anterior aspect of the preferred upper thigh and was fully wrapped in waterproof adhesive tape to allow participants to wear the activPAL3 during showering and bathing activities. The commercial manufacture’s software was used to initialise the accelerometer with the default settings (i.e., 20 Hz, 10s minimum sitting-upright period) and to process the recorded data (activPAL process and presentation V7.2.32). The algorithm detects postures (sedentary, standing, and walking), counting steps and sit-to-stand transitions. The activities detected can be classified as inactivity (that is time in a sedentary position, i.e. lying or sitting) and upright time, such as standing and walking [26]. PA was measured three times during the study: after admission to the geriatric inpatient rehabilitation (T0), which is usually the third week after the fracture, prior to discharge from the rehabilitation clinic (T1), and three months after discharge (T2). At T0 and T1 the first weekday with activity measurements over 24-h was included in the analysis. At T2 seven consecutive days (24 h each) were analysed and the mean values per day were presented. The sensor was attached the day before the start of each measurement period to ensure 24-h recordings for all measurement days. The rationale to limit the assessment at T0 and T1 to 24 h was the equal structure of days across the week during geriatric rehabilitation, set by the clinical routine. In contrast, a 7-day assessment was performed at T2 to account for the inter-day variability in the home-environment [27].

#### Global cumulative PA parameters

In the present study, we have followed the analyses previously described by Klenk and colleagues [28] analysing global cumulative PA parameters, such as the average daily walking duration, average daily number of steps and average daily upright duration.

#### In-depth PA parameters

As Klenk and colleagues [28] suggested, also more detailed ‘in-depth’ parameters to describe PA after hip fracture have been derived from these global cumulative parameters: average daily walking interval length, absolute number of walking bouts of different durations (≥1 s, ≥10 s, ≥60 s), median cadence, average daily number of sit-to-stand transfers, and the coefficient of variation for walking interval lengths ≥10s, which is defined as the ratio of standard deviation to the mean and reflects in our example the variation of longer walking intervals (high values mean high variation). Walking bout thresholds of 1, 10 and 60 s to count the number of bouts have been selected pragmatically to avoid unplausible short bout lengths and to differentiate between bouts more likely acquired indoors and outdoors. Each parameter was determined per 24 h.

### Physical capacity parameters

Habitual gait speed and 5-chair-rise time [29] were assessed in order to include gold standard PC parameters currently being used in clinical routine and reflecting dynamic balance and functional leg strength, respectively. Habitual gait speed was measured by stopwatch over a distance of 4 m with an additional meter at either end for acceleration and deceleration. The assessment of the 5-chair-rise time was modified as recommended [30] allowing the use of arm-rests if needed and performing with habitual speed.

### Covariables

Age and sex were ascertained by patient file. Education was asked by interview. Cognitive function was screened by the Short Orientation–Memory–Concentration Test (SOMC) [25], a test which contains six weighted items. Scores range from 0 to 28 points, with lower scores indicating low cognitive impairment. The Short Physical Performance Battery (SPPB) [29] was performed to assess the capacity of lower extremities. The SPPB was developed for community-dwelling older persons and combines the results of tests assessing static balance, strength of the lower extremities and gait speed. Due to our different target group some modifications were made to the original versions of the strength and gait speed subtests as described above. For calculating the final SPPB score (range 0–12 with higher scores indicating the highest degree of lower extremity capacity) we used categorical scores (range 0–4) for each sub-test based on timed quartiles in a large population available on the internet [31]. Mobility disability was measured using the Rivermead Mobility Index (RMI) [32, 33]. The Short Falls Efficacy Scale – International (Short FES-I) [34, 35] was used to assess concern about falls for 7 activities of daily living (e. g. getting dressed or undressed). It ranges from 7 to 28 points, with lower scores suggesting low concern. Pain during the rehabilitation was assessed with the dimension “pain” of the Western Ontario and McMaster Universities Osteoarthritis-Scale (WOMAC) [36, 37]. This sub-scale consists of 5 items referring to pain during movement and in rest. Pain is indicated on a 11-point Likert scale (0 to 10) with a higher value representing more pain.

### Statistical analyses

The analyses focused on the progression of global cumulative as well as ‘in-depth’ PA-parameters between times of measurement. Least-square means and mean differences with 95%-confidence intervals were calculated for each point in time. For comparison, the same analyses were applied to the PC measures gait speed and 5-chair-rise, traditionally being used in clinical routine. Statistical analyses were performed using SAS 9.4.

## Results

The study population comprised 111 individuals. Seventy-five percent of the participants were female, and the mean age was 82.5 years (SD = 6.76 years). Participants’ characteristics are presented in Table 1.

**Table 1:** Characteristics of the study population at admission (T0)

	Total *n* = 111
Females, n (%)	83 (75)
Age [years], mean (SD)	82.5 (6.76)
Education > 9 years, n (%)	45 (41)
SOMC [0–28], mean (SD)	3.28 (2.75)
Gait speed [m/s], mean (SD)^a^	0.45 (0.16)
5-Chair rise time [s], mean (SD)^a^	32.3 (15.1)
SPPB [0–16], mean (SD)	3.04 (2.04)
RMI [0–15], mean (SD)	7.56 (2.54)
Short FES-I [7–28], mean (SD)	15.8 (4.96)
WOMAC pain [0–20], mean (SD)	14.3 (10.7)

*SOMC* Short Orientation-Memory-Concentration Test, *SPPB* Short Physical Performance Battery, *RMI* Rivermead Mobility Index, *Short FES-I* Short version of the Falls Efficacy Scale – International, *WOMAC* Western Ontario and McMaster Universities Osteoarthritis-Scale

^a^Gait speed at baseline was assessed from *n* = 89, chair rise time at baseline was assessed from *n* = 85

### Global cumulative PA parameters

Average daily walking duration, average daily number of steps and average daily upright duration increased during inpatient rehabilitation and further until to the follow-up three months after discharge. Walking duration and number of steps had their main gain during geriatric rehabilitation (rise by 56% up to 35.0 min and by 64% up to 2141 steps, respectively). These parameters levelled off in the home environment (rise by 25% up to 43.7 min and by 34% up to 2872 steps, respectively). The increase of daily upright duration between T0 and T1 (+ 27%) and between T1 and T2 (+ 33%) were similar (Table 2). Over the whole rehabilitation period, walking duration, number of steps and upright duration rose by 94, 120 and 70%, respectively.

**Table 2:** Mean values and differences with 95% confidence intervals (95%-CI) of PA parameters and PC measures

	T0 (*n* = 111)	T0-T1	T1 (*n* = 103)	T1-T2	T2 (*n* = 92)
**Global cumulative PA parameters**					
Average daily walking duration [min]	22.5 (17.5; 27.4)	12.5 (9.2; 15.8)	35.0 (30.0; 40.0)	8.7 (2.3; 15.1)	43.7 (36.8; 50.6)
Average daily number of steps	1304 (1019; 1589)	837 (595; 1080)	2141 (1857; 2426)	731 (278; 1184)	2872 (2383; 3361)
Average daily upright duration [min]	171.9 (146.4; 197.5)	46.9 (27.8; 65.9)	218.8 (193.4; 244.2)	72.6 (45.1; 100.2)	291.4 (257.8; 325.0)
**In-depth PA parameters**					
Average daily walking interval length [s]	11.9 (10.6; 13.1)	2.34 (0.68; 4.00)	14.2 (12.7; 15.7)	−4.19 (−5.56; −2.82)	10.0 (9.2; 10.8)
Coefficient of variation for walking interval lengths ≥10s	0.70 (0.66; 0.75)	0.08 (0.02; 0.15)	0.79 (0.72; 0.85)	0.22 (0.11; 0.34)	1.01 (0.88; 1.14)
Number of walking bouts ≥1 s	99 (81; 116)	49 (38; 60)	147 (130; 165)	94 (64; 124)	241 (207; 276)
Number of walking bouts ≥10 s	36 (28; 43)	19 (14; 24)	54 (47; 62)	21 (11; 32)	76 (64; 88)
Number of walking bouts ≥60 s	4 (2; 5)	2 (1; 3)	6 (5; 7)	−2 (−4; − 1)	3 (3; 4)
Median cadence [steps/min]	52.6 (50.3; 54.9)	6.23 (4.05; 8.41)	58.8 (56.8; 60.8)	3.14 (−0.41; 6.68)	61.9 (58.6; 65.3)
Average daily number of sit-to-stand transfers	49 (45; 53)	7 (4; 10)	56 (52; 60)	−12 (−15; −8)	44 (41; 48)
**Traditional PC measures**					
Gait speed [m/s]^a^	0.45 (0.41; 0.48)	0.09 (0.06; 0.13)	0.54 (0.50; 0.58)	−0.01 (−0.06; 0.04)	0.53 (0.48; 0.58)
5-Chair rise time [s]^a^	34.3 (31.0; 37.6)	−5.09 (−7.69; −2.48)	29.2 (26.4; 32.1)	0.36 (−2.80; 3.52)	28.9 (25.8; 31.9)

*T0* at admission, *T1* at discharge, *T2* three months after discharge

^a^ Measured by stop-watch

### In-depth PA parameters

Similarly, to the cumulative PA measures, the total number of walking bouts ≥1 s continuously rose over the whole observation period by 143% (from 99 to 241 bouts), but mainly after discharge with an increase of 64%. The number of walking bouts ≥10 s rose by 111% (from 36 to 76 bouts) in total with equal slopes in both periods. In contrast, the number of longer bouts ≥60 s increased during inpatient rehabilitation by 50% (+ 2) and decreased by the same amount at home. All changes were statistically significant as indicated by the confidence intervals of the mean value differences.

The observed pattern for number of walking bouts was also reflected by the daily walking interval length with an increase by 2.34 s (95% confidence interval (CI): 0.68; 4.00) from 11.9 s to 14.2 s during inpatient rehabilitation. However, the walking interval length decreased after discharge even below the baseline level to 10.0 s (− 4.19 s (95% CI: − 5.56; − 2.82). The variability for walking interval lengths ≥10s indicated by the coefficient of variation increased slightly between T0 and T1 by 13% and more pronounced between T1 and T2 by 28%.

Median cadence only statistically significantly improved during inpatient rehabilitation by 6.23 steps per minute (95% CI: 4.05; 8.41). The average daily number of sit-to-stand transfers increased during geriatric rehabilitation by 14% (49 to 56 sit-to-stand transfers), but decreased in the home environment below the baseline level (44 sit-to-stand transfers).

### Physical capacity parameters

The PC in-lab measures of gait speed and 5-chair rise time showed a comparable pattern to the in-depth PA parameters with a statistically significant improvement during inpatient rehabilitation and no further progression after discharge.

## Discussion

Until now, only a few studies on fragility fracture patients have reported objective sensor-based PA measures. These have mainly analysed global cumulative parameters, such as walking duration, uptime and step counts. In our study we additionally examined further in-depth PA parameters. These parameters showed different patterns and obtain a more detailed picture of mobility during rehabilitation and after discharge, which might better reflect different clinically relevant domains of PA, such as PC. The in-depth PA parameters seem also to have been affected by behavioural and environmental factors.

All measured global cumulative PA parameters assessed by an inertial sensor improved statistically significantly and clinically meaningfully during inpatient rehabilitation and after discharge. At home uptime duration (standing, shuffling, walking) reached more than 4 h a day. Other published studies showed improvements in global cumulative PA parameters such as “daily walking duration”, “daily number of steps” and/or “daily upright time” [20, 38, 39]. The significant improvement of daily walking duration and the number of steps during geriatric inpatient rehabilitation is likely to be caused by the rehabilitation and the build environment of the rehabilitation clinic. Patients received at least three single or group sessions of physiotherapy, weight-bearing exercise and balance training. The different slopes of the three measured global cumulative PA parameters indicate a shift from walking to standing activities after discharge. This is most likely caused by a behavioural adaptation to the environment and social role. At home many people get involved in standing household activities, such as preparing a meal, washing the dishes and cleaning the apartment [40]. Future interventions for fragility fracture patients might focus more on the training of outdoor activities to increase the individual live-space and thereby social participation and quality of life.

The in-depth PA parameters underpin the change of the global cumulative PA parameters after discharge, but give further insights in to the change of PA patterns. The following three findings underline the shift to shorter walking bouts after discharge: the decrease of the mean walking interval length after an initial increase during rehabilitation, the rising number of walking bouts and the decline of longer bouts ≥60 s at home. The profiling shows that after discharge patients are longer on their feet, but interrupt walking activities more often to do other standing activities. This probably reflects the required person-environment fit. The walking bouts of 10 s and more are particularly relevant for longer distance indoor activities such as transfers between rooms at home. In contrast, walking bouts of 60 s and more reflect very long indoor distances like in the inpatient setting (e.g., transfer from a patient room to a therapy room) or outdoor activities. The majority of the participants in the study were mostly homebound, which corresponds to the observed decrease of walking bouts of 60 s and more. Somewhat similar observations have been reported by other publications in the field [41]. The increase of the coefficient of variation for walking interval lengths over 10 s particularly after discharge demonstrates a higher variability of longer walking intervals and therefore a higher adaptability to activities of daily living. The number of sit-to-stand transfers increased during inpatient rehabilitation but decreased below baseline level after discharge. The most likely explanation is the content of therapy sessions. In most physiotherapy sessions patients are trained to get on and off a chair and also during the standing group exercises patients often sit down and get off the chair again. This could be seen as a potential to monitor part of the therapy sessions using inertial sensors.

The current clinical gold standard PC parameters gait speed and chair rise improved by 10–20% during inpatient rehabilitation, but not after discharge. The pattern corresponds to the progress of the in-depth PA parameters median cadence and number of sit-to-stand transfers, which might reflect the same PA domain, namely PC. These in-depth PA parameters could help to continuously monitor PC during rehabilitation and at home. Our results indicate that outpatient rehabilitation measures are lacking in this population. Outpatient programmes such as the Eva-Hip study [42] show that gait recovery after hip fracture can be further improved after discharge by a home-based balance and gait exercise programme.

Our findings suggest that the in-depth PA parameters add further information to the global cumulative PA measures. Whereas all parameters improved significantly during inpatient rehabilitation, some parameters, which might be mainly related to PC, did not continue to improve after discharge. Furthermore, contextual factors such as the build environment probably have an influence on PA parameters. Therefore, we think that a matrix of in-depth digital mobility parameters can add important information to better describe domains of PA, such as PC, behavioural and environmental factors, and its changes. However, the validity of these in-depth PA parameters has to be proven in future studies.

We have to acknowledge certain limitations. First, it has been shown that activPAL and other sensors [43] may underestimate step counts during slow walking. As walking speed at T0 was only 0.45 m/s, the number of steps and daily walking duration at T0 may have been underestimated and hence, the difference between T0 and T1 could have been lower than assumed. Second, walking bouts ≥1 s might also include data which do not reflect walking. However, the commercial manufacture’s software provides these bout lengths. Therefore, we decided to present the number of bouts in relation to different cut points for the minimum bout length (1 s, 10s, 60s). A third limitation is the lacking ground truth for some of the assumptions. It was not possible to validate the contextual meaning of some of the parameters. The walking bout duration is likely to reflect in-room, between-rooms, and outdoor activities. To test this hypothesis, it requires either validation by direct observation or technical validation such as automatic beacon labelling and geo-location tracking. Furthermore, it was not possible to differentiate between therapeutical and habitual activity during the inpatient period. Activity during rehabilitation was partly externally controlled by therapy sessions. Also, further factors such as the build environment (e.g., room location, corridor design) and social contacts by family and peers are likely to influence PA as co-variates or confounders. There is a need of further studies to investigate these aspects and to directly link observed PA patterns to certain components of capacity, environment and behaviour. By definition it is not possible for the study to conclude that inpatient rehabilitation alone was responsible for the beneficial effect on PA observed.

## Conclusions

We observed the changes of PA patterns in patients after hip and pelvic fracture during inpatient rehabilitation and transition to home settings. Global cumulative PA parameters, such as walking duration, number of steps and upright duration provide measures of total activity. The in-depth PA parameters add more detailed information about different domains of PA: They seem to reflect the living environment and aspects of PC, which are traditionally assessed by PC measures, such as gait speed or 5-Chair rise time. In combination with these classical clinical outcomes, digital mobility measures can help clinicians to assess the health status of fragility fracture patients, individually tailor therapy measures and monitor the rehabilitation process.

## Data Availability

The datasets used and/or analysed during the current study are available from the corresponding author on reasonable request.

## Abbreviations

PA: Physical activity
PC: Physical capacity
CI: Confidence interval
Short FES-I: Short Falls Efficacy Scale – International
